# Tularemia in Children, Turkey, September 2009–November 2012 

**DOI:** 10.3201/eid2101.131127

**Published:** 2015-01

**Authors:** Hasan Tezer, Aslınur Ozkaya-Parlakay, Hakan Aykan, Mustafa Erkocoglu, Belgin Gülhan, Ahmet Demir, Saliha Kanik-Yuksek, Anil Tapisiz, Meltem Polat, Soner Kara, Ilker Devrim, Selcuk Kilic

**Affiliations:** Gazi University School of Medicine, Ankara, Turkey (H. Tezer, A. Tapisiz, M. Polat, S. Kara);; Ankara Hematology Oncology Children’s Training and Research Hospital, Ankara (A. Ozkaya-Parlakay, H. Aykan, M. Erkocoglu, B. Gülhan, A. Demir, S. Kanik-Yuksek);; Dr. Behcet Uz Children's Training and Research Hospital, Izmir, Turkey (I. Devrim);; Public Health Institution of Turkey, Ankara (S. Kilic)

**Keywords:** tularemia, children, oropharyngeal form, treatment, relapse, bacteria, zoonoses

## Abstract

Tularemia, a zoonotic disease caused by *Francisella tularensis*, is found throughout most of the Northern Hemisphere. It is not well known and is often misdiagnosed in children. Our aim with this study was to evaluate the diagnosis, treatment, and prognosis for 100 children with tularemia in Turkey. The mean patient age was 10.1 ± 3.5 years (range 3–18 years), and most (63%) patients were male. The most common physical signs and laboratory findings were cervical lymphadenopathy (92%) and elevated erythrocyte sedimentation rate (89%). Treatment response was higher and rate of relapse lower for children 5–10 years of age than for those in other age groups. Associated with treatment failure were female sex, treatment delay of ≥16 days, and use of doxycycline. Tularemia is endemic to Turkey, and the number of cases has been increasing among children as well as adults.

## Abstract 2

Clinical course in children differs from that in adults.

Tularemia, caused by *Francisella tularensis*, is a potentially fatal, multisystemic disease in humans. Tularemia occurs throughout most of the Northern Hemisphere, and the number of cases is increasing in various parts of Europe, especially in the Balkans, Turkey, and Scandinavian countries. There are 4 recognized subspecies of *F. tularensis*, which differ in their pathogenicity and geographic distribution: *tularensis* (type A), *holarctica* (type B), *novicida*, and *mediasiatica*. Among them, subspecies *tularensis* and *holarctica* are of particular clinical and epidemiologic relevance (*1*–*4*). Although the highly virulent subspecies *tularensis* is restricted almost exclusively to North America, subspecies *holarctica* is found in Europe, Asia, and North America and represents the most common subspecies involved in human and animal infection (*4*).

The clinical forms of tularemia are ulceroglandular or glandular, oculoglandular, oropharyngeal, respiratory, and typhoidal (*1*). Each form somehow reflects the mode of transmission. The clinical picture and severity of the disease in humans vary considerably depending on the route of infection, the virulence of the causative organism, and the immune status of the host. The ulceroglandular form has been reported as the most prevalent clinical form of the disease in northern Europe, whereas the oropharyngeal form has been most commonly reported in Turkey, Bulgaria, and Kosovo and is attributed to the consumption of contaminated water and food (*5*–*10*).

Tularemia is endemic to Turkey, and most cases are reported to occur in late summer or early autumn (*10*). Various studies on clinical course, treatment, and treatment failure in elderly patients are available in the literature (*7*,*10*–*12*). However, the clinical course of tularemia in children is not well known, and cases in children are often misdiagnosed. Our aim was to demonstrate the clinical features and outcomes for children with tularemia.

## Material and Methods

We conducted a retrospective records review for 100 children with a presumptive diagnosis of tularemia who were admitted to the Ankara Hematology Oncology Children’s Training and Research Hospital and Gazi University Hospital from September 2009 through November 2012. The diagnosis of tularemia was confirmed by detection of specific antibodies by microagglutination test and/or *F. tularensis* nucleic acid in a clinical specimen (lymph node aspirate).

Microagglutination testing was performed as described (*1*) by using safranin-stained *F. tularensis* cells from the National Collection of Type Cultures (*F. tularensis* NCTC 10857, *F. tularensis* subsp. *holartica* live vaccine strain) cells. In brief, 2-fold serial dilutions of serum were incubated overnight with safranin-stained, formalin-killed *F. tularensis* cells at 35°C, and a titer was assigned according to the last well demonstrating full agglutination. According to the legal health regulations in Turkey, a diagnosis of tularemia is serologically confirmed by the presence of at least 1 of the following laboratory findings: 1) compatible clinical signs or symptoms and specific antibodies at significantly high titers (>1:160) and 2) a >4-fold increase in 2 successive titers (>1:160 for the convalescent-phase titer). Therefore, antibody titers >1:160 were considered to be positive.

DNA from clinical samples was extracted by using a commercial purification system with columns (QIAamp DNA extraction mini kit; QIAGEN GmbH, Hilden, Germany) according to the manufacturer’s instructions. Clinical samples from patients with suspected tularemia were screened for evidence of the tularemia agent by using conventional PCR.

Affliation with the genus *Francisella* was confirmed by amplification of the 17-kDa outer membrane lipoprotein gene fragment as described by Sjöstedt et al. (*13*). Primers TUL4–435 (5′-GCT GTA TCA TCA TTT AAT AAA CTG CTG-3′) and TUL4–863 (5′-TTG GGA AGC TTG TAT CAT GGC ACT-3′), which amplify a 420-bp fragment of the 17-kDa lipoprotein, were used. Samples were added to a PCR mixture containing a final concentration of 200 mmol/L (each) deoxynucleoside triphosphate mixture, 10X Hot Start PCR Buffer (Fermentas/Thermo Scientific, Vilnius, Lithuania), 0.4 mmol/L (each) primer (Ella Biotech GmbH, Martinsried, Germany), 2.5 mmol/L MgCl_2_ (Fermentas/Thermo Fisher Scientific), 1 μL bovine serum albumin (1 mg/mL, Sigma-Aldrich), and 1.25 U *Taq* DNA polymerase (Fermentas/Thermo Fisher Scientific) in a total reaction volume of 50 μL. The reaction was performed in a DNA thermal cycler (Thermo Hybaid OMN-E, Ashford, UK) at a denaturation temperature of 94°C for 4 min and was followed by 40 cycles at 94°C for 40 s, 64°C for 30 s, and 72°C for 45 s, and final extension at 72°C for 5 min. The PCR products were sized on agarose gels and stained with ethidium bromide.

After confirmation of isolates as *F. tularensis* by PCR with *tul*4 primers, conventional PCR selective for the region of difference – 1 was used to determine subspecies identity (*14*). PCR for detection of *Mycobacterium tuberculosis* complex was also performed on lymph node aspirates. Patients with abdominal pain were evaluated by abdominal ultrasonography.

Treatment options varied according to the patient’s age. One of the following regimens was administered: gentamicin (5 mg/kg 2 or 3 times daily, intravenously or intramuscularly) for 10 days, doxycycline (100 mg orally 2×) for 14 days, ciprofloxacin (20 mg/kg 2×) for 10–14-days, or streptomycin (30–40 mg/kg/day, divided into 2 doses/day, intramuscularly) for 10 days. Doxycycline was not the drug of choice for patients younger than 8 years.

Good response was defined as complete recovery from the illness after treatment with antimicrobial drugs only (no suppuration, no need for surgical procedures, and no relapse). Therapeutic failure was defined as the presence of 1 of the following: increased lymph node size, occurrence of new lymphadenopathy, or both; persistent or recurrent fever; and constantly elevated or increasing acute-phase reactants (erythrocyte sedimentation rate and C-reactive protein levels).

Statistical analyses were performed by using SPSS version 20 (SPSS Inc., Chicago, IL, USA). Numerical variables are expressed as means (or medians) and standard deviations. Categorical variables are expressed as frequencies and percentages. For categorical variables, if the χ^2^ condition was met, multiple group comparisons were performed by χ^2^ test; if not, Monte Carlo simulation was used in multiple group comparisons and the Fisher exact test was used to compare 2 groups. Numerical variables with normal distribution were compared by using the Student *t*-test, and those with nonnormal distribution were compared by using the Mann–Whitney U test. Logistic regression analysis (method = ENTER) was used to determine the risk factors. A p value <0.05 was considered to be significant.

## Results

**Figure 1:**
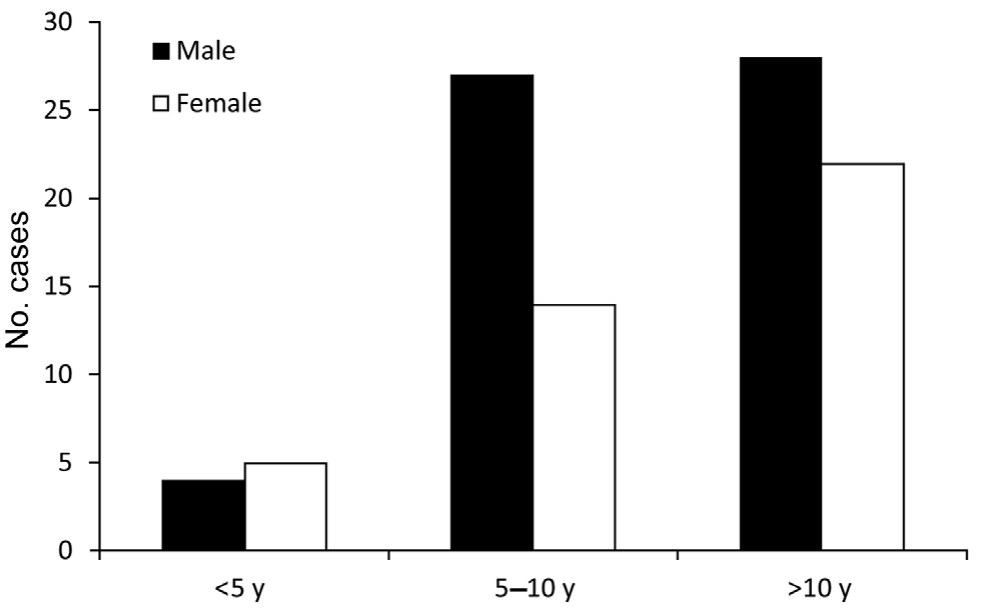
Diagnosis of tularemia for 100 children, by patient age group and sex, Ankara, Turkey, September 2009–November 2012.

**Figure 2:**
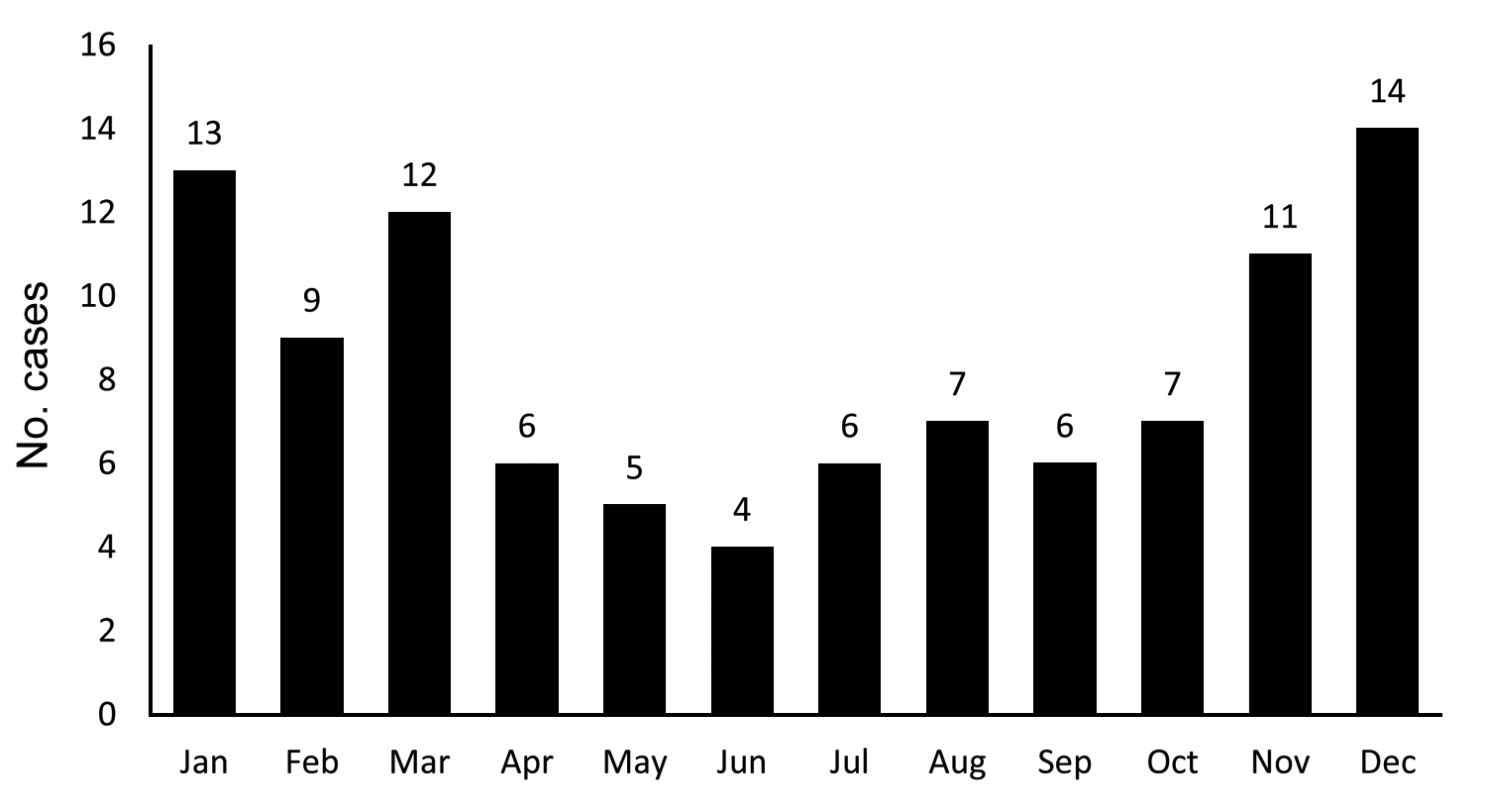
Diagnosis of tularemia for 100 children, by month, Ankara, Turkey, September 2009–November 2012.

A total of 100 children with laboratory-confirmed tularemia were included in the study. Most (63%) of the children were male. Mean patient age was 10.1 ± 3.5 years (range 3–18 years); half (50%) were younger than 10 years, and only 9% were younger than 5 years (Figure 1). Most (96%) patients were from Ankara Province, and only 4% were from the Aegean region. Most (62%) patients lived in villages in Ankara, and the rest (16%) came from an area ≈250 km in diameter. More than half (60%) of the patients were admitted to the hospital during winter and autumn (Figure 2). In terms of risk factors, in addition to tap water, natural stream water was available and had been widely used for drinking and cooking; 76% of the patients had a history of drinking water from these sources. Contact with rodents was reported for 32% of patients, and 44% of patients had at least 1 neighbor who had been exposed to rodents. None of the patients had a history of tularemia.

### Clinical Findings 

The most common clinical signs were swelling on the neck (cervical lymphadenopathy) (92%), fever (63%), and tonsillitis (55%) (Table 1). Abdominal lymphadenopathy was found in 3 patients. Oropharyngeal tularemia was diagnosed for 90 patients, ulceroglandular tularemia for 7, and oculoglandular tularemia for 3 (Table 1). No patient died during or after therapy.

**Table 1:** Clinical characteristics of 100 children with tularemia, Ankara, Turkey, September 2009–November 2012*

Characteristic	No. children
Clinical sign	
Cervical lymphadenopathy	92
Unilateral	73
Bilateral	19
Abdominal lymphadenopathy	3
Tonsillitis	55
Fever	63
Conjunctivitis	5
Ulceration	5
Diarrhea	4
Erythema nodosum	4
Disease form	
Oropharyngeal	90
Ulceroglandular	7
Oculoglandular	3

*Mean patient age was 10.1 ± 3.5 years; 63 boys, 37 girls.

During the initial visits, skin rashes were found on 6 patients; 4 of these patients had papular lesions on the extremities, and the other 2 had erythema nodosum without associated symptoms of tularemia. During follow-up visits, 1 of the patients with erythema nodosum had oculoglandular tularemia and the other had oropharyngeal tularemia.

### Laboratory Findings

Diagnoses were primarily established by microagglutination test results (titer >1:160) for 98 patients and by *F. tularensis*–specific PCR analysis for 2 patients. The microagglutination method revealed titers of 1:40–1:2,560. The median agglutinin titer was 1:810. Paired serum samples from 2 patients with low acute-phase titers revealed a 4–fold increase in titer.

The other abnormal laboratory findings were elevated leukocyte counts and erythrocyte sedimentation rates. Mean leukocyte count was 11,918 ± 3,924/mm^3^ (range 5,600–23,800/mm^3^). Erythrocyte sedimentation rate was elevated for 89% of the patients (mean 60.13 ± 27.5 mm/h, range 8–120 mm/h), and for 68% of patients the rate was >50 mm/h. For 18 patients, only C-reactive protein was elevated (median 2.51 mg/dL, range 0.1–19.2 mg/dL). Ultrasonography was performed for 89 patients with lymphadenopathy and for 77 of these patients revealed cystic necrotic abscesses characterized by central hypoechogenicity and septations. No significant difference was found between erythrocyte sedimentation rate (>50 mm/h vs. <50 mm/h) and C-reactive protein levels, respectively, of patients with good response (p = 0.133, p = 0.819), those who needed surgical procedures (p = 0.131, p = 0.103), those who experienced spontaneous suppuration (p = 0.448, p = 0.674), and those who experienced relapse (p = 0.325, p = 0.963).

### Treatment Response

No deaths occurred during the 12-month follow-up period. Of 62 patients in the treatment failure group, previous medications included β-lactams/macrolides. Another 2 patients had received a diagnosis of tuberculosis according to pathologic findings and were receiving antituberculosis treatment at the time of diagnosis.

Initial therapy consisted of intravenous gentamicin (56 patients), oral doxycycline (23 patients), oral ciprofloxacin (20 patients), or intramuscular streptomycin (1 patient). The duration of antimicrobial drug treatment was 10–14 days. Relapse rates were similar for patients who received gentamicin, doxycycline, or ciprofloxacin (p = 0.306).

**Figure 3:**
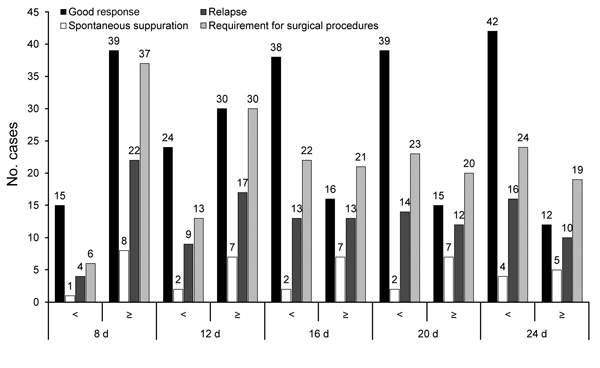
Data regarding outcome for 100 children with tularemia, Ankara, Turkey, September 2009–November 2012.

Complete recovery with no complications (e.g., suppuration, need for surgical procedures, or relapse) occurred for 54 patients. However, for 8 patients with a good response, although surgery was not needed, a surgical procedure was performed to hasten the healing process. While receiving specific antimicrobial drug therapy, 9 patients experienced spontaneous suppuration. During the 12-month follow-up period after initial treatment (antimicrobial drug alone or with surgical procedures), 74 patients neither required re-treatment nor experienced relapses. A surgical procedure was performed for 43 (58.1%) of these 74 patients. The remaining 26 patients experienced relapses that required a second course of therapy (Table 2). All 26 patients received antimicrobial drugs, and 17 underwent surgical procedures. Among these 17 patients, 10 underwent a second surgical procedure. All 17 patients recovered completely and did not experience any relapses during the 12-month follow-up period. Outcome data are shown in Figure 3. Excisional biopsy specimens from 15 patients underwent histopathologic examination and revealed chronic necrotizing lymphadenitis with histiocytic infiltration and caseous necrosis.

**Table 2:** Outcomes for 100 children with tularemia, Ankara, Turkey, September 2009–November 2012

Variable	Treatment response, no. (%) children

Good response, n = 54	Spontaneous suppuration, n = 9	Relapse, n = 26	Needed surgical procedure, n = 43
Age group, y				
<5, n = 9	4 (44.4)	1 (11.1)	4 (44.4)	3 (33.3)
5–10, n = 41	29 (70.7)	2 (4.9)	5 (12.2)	15 (36.6)
>10, n = 50	21 (42)	6 (12)	17 (34)	25 (50)
p value	p = 0.02	p = 0.46	p = 0.03	p = 0.36
Treatment delay, d				
<16, n = 60	38 (63.3)	13 (21.7)	2 (3.3)	22 (36.7)
>16, n = 40	16 (40.0)	13 (32.5)	7 (17.5)	21 (52.5)
p value	p = 0.02	p = 0.23	p = 0.03	p = 0.12

### Factors Predicting Response to Treatment

#### Age

Complete recovery rate (good response) was higher among children 5–10 years of age than among those in the other age groups (<5 and >10 years) (p = 0.018) (Table 2). The need for surgical procedures (p = 0.36) and spontaneous suppuration of lymph nodes (p = 0.46) were similar for children in all age groups. Rates of relapse were significantly lower for children 5–10 years of age than for those in the other age groups (p = 0.03) (Table 2).

#### Treatment Delay 

For 56 patients, the time from diagnosis to treatment initiation was >2 weeks. Good response rate (p = 0.02) was significantly lower and relapse rate was significantly higher (p = 0.03) among patients for whom treatment was delayed >16 days, and spontaneous suppuration rate (p = 0.23) and need for surgical procedures (p = 0.12) were similar among patients for whom treatment was delayed >16 days and those for whom treatment was delayed <16 days (Table 2).

According to regression analysis, significant factors for treatment failure were female sex, treatment delay >16 days, and doxycycline use. The significant factor for spontaneous suppuration was treatment delay >16 days (Table 3).

**Table 3:** Results of regression analysis for variables predicting treatment outcomes for 100 children with tularemia, Ankara, Turkey, September 2009–November 2012*

Variable	Treatment failure		Spontaneous suppuration		Relapse		Requirement for surgical procedures

OR (95% CI)	p value	OR (95% CI)	p value	OR (95% CI)	p value	OR (95% CI)	p value
Female sex	3.449 (1.250–9.514)	0.017		1.348 (0.313–5.804)	0.689		2.314 (0.850–6.300)	0.101		1.842 (0.716–4.742)	0.205
Treatment delay >16 d	3.277 (1.220–8.799)	0.019		6.074 (1.185–31.131)	0.030		1.295 (0.477–3.517)	0.612		1.876 (0.741–4.751)	0.184
Antimicrobial drug											
GEN†		0.067		ND	ND			0.643			0.140
DOX	4.965 (1.280–19.264)	0.021		ND	ND		1.295 (0.355–4.733)	0.695		3.308 (0.957–11.428)	0.059
CIP	2.637 (0.733–9.483)	0.138		ND	ND		0.542 (0.124–2.371)	0.416		2.689 (0.808–8.950)	0.107
GEN + DOX	0.458 (0.060–3.473)	0.450		ND	ND		0.376 (0.035–4.033)	0.419		0.636 (0.094–4.317)	0.643
Age at diagnosis	0.926 (0.832–1.031)	0.162		0.941 (0.755–1.173)	0.589		0.925 (0.801–1.067)	0.283		0.935 (0.841–1.041)	0.219
Age at study inclusion	2.611 (0.739–9.222)	0.136		2.096 (0.151–29.018)	0.581		2.588 (0.476–14.086)	0.271		2.321 (0.658–8.183)	0.190

*Because the number of patients was limited, antimicrobial drugs were not included in the regression analysis for spontaneous suppuration. CIP, ciprofloxacin; DOX, doxycycline; GEN, gentamicin; ND, not done. †Reference variable.

## Discussion

Tularemia is endemic to Turkey. Although most studies have examined adult populations, tularemia can occur in patients of all ages and is more prevalent among children 5–9 years of age and in persons older than 75 years (*6*,*10*–*12*,*15*–*17*). Ulceroglandular tularemia is the most commonly reported clinical form throughout the world (*1*,*4*,*5*,*18*). However, prevalence of oropharyngeal tularemia is higher in Turkey, Bulgaria, Kosovo, and Norway (*6*,*7*,*10*,*15*–*17*,*19*), as is reported here.

In studies reported from Turkey, cases of tularemia in patients younger than 10 years were extremely rare; in a report of 61 tularemia cases, no patient was younger than 10 years, and in another study, only 2 patients were younger than 10 years (*11*,*17*). In our study, half of the patients were younger than 10 years. The prevalence of tularemia among children probably tended to increase because of increased exposure of farmers’ infants and school-aged children to farm animals, rodents, or rodent excreta while helping their families with farming and drinking nonchlorinated (natural spring) water.

The source of infection and the mode of transmission often remain elusive for patients with tularemia. However, the high prevalence of oropharyngeal tularemia has been associated with the consumption of contaminated water (*6*,*10*–*12*). Tularemia outbreaks that are associated with the consumption of hunted animals usually occur in summer and early autumn (*1*,*3*–*5*), whereas waterborne tularemia outbreaks usually occur in autumn and winter (*10*–*12*,*20*–*22*). Similar to cases in adults, almost all of the cases in children in our study occurred in winter or autumn.

*F. tularensis* subsp. *holarctica* usually causes mild infections with low mortality rates. For patients with tularemia, physical examination usually reveals unilateral cervical lymphadenopathy, tonsillitis, and fever. The most commonly reported finding in tularemia patients is lymphadenopathy (*6*,*11*,*12*,*22*). In our study, the most common (92%) complaint at admission was a mass in the neck region (cervical lymphadenopathy), followed by tonsillitis and fever.

Secondary skin manifestations of tularemia are erythema multiforme, erythema nodosum, and papular lesions. Skin manifestations might occur in all forms of tularemia and probably result from the systemic spread of organisms (*23*,*24*). In our study, erythema nodosum was observed in only 2 (2.6%) patients, less than that observed in other studies (*6*–*9*,*11*,*15*). Of note, none of the patients had any signs or symptoms suggesting tularemia at illness onset. In a study of 50 patients in Finland, the frequency of skin lesions was ≈50% (*25*). In our study, the frequency of skin lesions was as low as 6%.

Routine peripheral blood counts do not provide a diagnostic clue for tularemia. The standard test for diagnosis of tularemia is culture, which is difficult and requires a Biosafety Level 3 facility (*1*). Therefore, other methods, mainly serologic testing and PCR, have generally been preferred for the diagnosis of this disease (*26*–*29*). The most frequently ordered diagnostic testing for *F. tularensis* infection is serology. In Turkey, antibody titers of >1:160 obtained by microagglutination testing are generally consistent with infection (*6*,*10*–*12*,*21*,*30*). However, antibody levels do not usually increase until the second week of illness (*10*,*30*). In addition, the existence of antibody-negative but culture-positive patients has been reported in the literature (*30*). In another study, for some tularemia patients with negative microagglutination test results, diagnosis was made only by ELISA or PCR (*28*). In our study, microagglutination titers were >1:160 for 98 patients, and PCRs were positive for 2 patients with negative serologic test results. In the literature, the sensitivity and specificity of microagglutination testing, based on the results obtained for samples from healthy persons and patients with illnesses other than tularemia, were 97.6% and 98.7%, respectively (*30*).

In our study, 92 patients had cervical lymphadenopathy. Despite negative microagglutination test results, PCRs for *F. tularensis* in the tissue were positive. The patients recovered fully after taking antimicrobial drugs specific for tularemia. Thus, in our opinion, although microagglutination testing had been recommended for the diagnosis of tularemia, PCR testing should be considered for patients with signs and symptoms strongly suggestive of tularemia and with negative microagglutination test results.

PCR testing has been performed on samples (lymph node aspirates) collected during epidemics in Turkey (2009–2012) (*12**,**31**,**32*). The sensitivity of PCR ranged from 33.9% to 91.0% for patients with culture- and/or serology-verified oropharyngeal and glandular tularemia, depending on the duration of illness, and PCR was more sensitive than culture of lymph node aspirates (63.3% vs. 23.3%) (*12**,**31**,**32*). PCR is the most rapid and sensitive test available for detecting *F. tularensis* in patient specimens (*26*).

Beta-lactams, cephalosporins, and clindamycin are considered to be ineffective for the treatment of *F. tularensis* infections (*5*,*6*). In the United States and Europe, treatment with aminoglycosides has been the mainstay of treatment for children with tularemia (*3*,*33*,*34*). Because of the possible ototoxic side effects of aminoglycosides, evaluation of hearing function before treatment and monitoring of inner ear function during treatment are highly recommended (*1*,*11*). Expert groups in Europe recommend quinolones as an effective treatment alternative for subsp. *holarctica* infections (*29*,*33*). In our study, ciprofloxacin was preferred for 26 patients. No relapses occurred in these patients, although our sample size was not large enough to enable us to draw definitive conclusions. Further randomized studies in large patient populations are needed. Tetracyclines should be administered for at least 14–21 days because relapses in patients receiving these bacteriostatic agents have been reported (*11*). In patients younger than 8 years, the lack of alternative oral medications for tularemia constitutes a major problem. A study that evaluated 67 cases in children established that the time from symptom onset to diagnosis was 26 days (range 8–92 days). Delayed treatment was explained by the use of antimicrobial drugs not effective against *F. tularensis* in at least 20 patients. For some patients, although tularemia was suspected, treatment delays were attributed to lack of oral alternatives to aminoglycosides (*34*). Duration of treatment depends on the clinical course of the disease. In our study, the relapse rates were similar among patients receiving each of the 4 antimicrobial drugs, including doxycycline.

Delayed initiation of antimicrobial therapy was reported to be strongly associated with treatment response. In a recent study from Turkey that included adults, initiation of therapy after 14 days was reported to be associated with treatment failure (*29*) as it was in previous reports (*9*). In our study, factors that significantly affected treatment failure were female sex, treatment delay >16 days, and doxycycline use.

Recently a small-scale study from Turkey, which included children, reported that all children responded well to treatment regardless of age (*17*). However, in our study, response to treatment was better and rates of relapse were lower among children 5–10 years of age than among children in other age groups. This finding may be the target of immunopathogenesis research.

In conclusion, our study indicated that tularemia affects children differently than adults, suggesting that tularemia should be kept in mind as a diagnosis for children with severe lymphadenopathy and tonsillitis who show no response to β-lactam antimicrobial drugs. Additionally, according to our results, prognosis varied by age, and treatment failure was associated with female sex, treatment delay >16 days, and doxycycline use.
